# Evidence of the hydrogen release mechanism in bulk MgH_2_

**DOI:** 10.1038/srep08450

**Published:** 2015-02-13

**Authors:** Kazuhiro Nogita, Xuan Q. Tran, Tomokazu Yamamoto, Eishi Tanaka, Stuart D. McDonald, Christopher M. Gourlay, Kazuhiro Yasuda, Syo Matsumura

**Affiliations:** 1 Nihon Superior Centre for the Manufacture of Electronic Materials, School of Mechanical and Mining Engineering, The University of Queensland, Brisbane, QLD 4072, Australia; 2 Department of Applied Quantum Physics and Nuclear Engineering and the Ultramicroscopy Research Center, Kyushu University, Fukuoka 819-0395, Japan; 3 Department of Materials, Imperial College, London. SW7 2AZ. UK

## Abstract

Hydrogen has the potential to power much of the modern world with only water as a by-product, but storing hydrogen safely and efficiently in solid form such as magnesium hydride remains a major obstacle. A significant challenge has been the difficulty of proving the hydriding/dehydriding mechanisms and, therefore, the mechanisms have long been the subject of debate. Here we use in situ ultra-high voltage transmission electron microscopy (TEM) to directly verify the mechanisms of the hydride decomposition of bulk MgH_2_ in Mg-Ni alloys. We find that the hydrogen release mechanism from bulk (2 μm) MgH_2_ particles is based on the growth of multiple pre-existing Mg crystallites within the MgH_2_ matrix, present due to the difficulty of fully transforming all Mg during a hydrogenation cycle whereas, in thin samples analogous to nano-powders, dehydriding occurs by a ‘shrinking core' mechanism.

The storage of hydrogen in solid form as magnesium hydride is a promising technology that is being developed for fuel cells for automotive and stationary applications and to enable safer hydrogen transportation^1^,^2^,^3^. Pure magnesium has a hydrogen carrying capacity of around 7.6 wt%, however, the resulting hydride is relatively stable and the temperature must be increased to 300°C at 1 bar H_2_ for the hydrogen to be released with acceptable release rate with realistic industry use^2^. Design goals for on-board storage (automotive) aim for a temperature of hydrogen release of between 60 and 120°C^2^, however land-based consumer and industrial storage systems do not have such stringent requirements^4^. Much research on Mg-based H-storage materials is based on ball milled nano-scale powders due to their superior H-sorption properties over bulk powders^2^,^5^. Another approach is to improve the H-sorption properties of bulk powders, for example, by treating liquid Mg-alloys with alkali and alkali-earth metals prior to casting and powder production^6^. A significant challenge in research on both nano-powders and bulk-powders has been the difficulty of proving the hydriding/dehydriding mechanisms and, therefore, the mechanisms have long been the subject of debate^1^,^2^,^3^. Multiple models for the desorption of MgH_2_ have been developed and these have been placed in several categories including ‘shrinking core' models^3^,^7^,^8^,^9^, ‘nucleation and growth' models^3^,^7^,^8^,^9^, ‘multiple step' kinetic models^3^,^7^,^8^,^9^, ‘migration and coalescence' (Greenwood and Speight) model^10^, and an Ostwald ripening model, eg. Ref. 11. Of these models the first two contain largely opposing ideas yet both approaches have been used with some success in the modeling of experimental results from either pressure-composition-temperature (PCT) curves or thermal gravimetric analysis (TGA) and differential thermal analysis (DTA) data^3^. These techniques and others including in-situ heating X-ray diffraction (XRD)^2^,^12^ and room temperature TEM^13^,^14^,^15^,^16^ do not give direct evidence of the operating mechanisms. Such evidence requires 4-D data, ie., three dimensional observations over time. As such, in-situ temperature controlled TEM observation is an attractive approach for determining the mechanism of hydrogen release. Three groups have reported using in-situ TEM at 200 kV to observe hydrogen desorption^15^,^17^,^18^. However, conventional TEM with an accelerating voltage of 200 kV has disadvantages including inelastic incident beam interactions with the samples, and the sample dimensions (typically less than 100 nm in thickness) make surface effects more prominent^19^. Recently Mooij and Dam^20^ demonstrated in-situ the two dimensional nucleation and growth of single hydride domains of up to several millimeters in diameter by an optical transmission technique (hydrogenography) for thin Mg film. They found that the nucleation and growth process affects the hysteresis between absorption and desorption.

Here we show the hydrogen release behavior from MgH_2_ in real-time using in situ ultra-high voltage transmission electron microscopy (TEM), and directly verify the mechanisms of the hydride decomposition of bulk MgH_2_ in Mg-Ni alloys. The ultra-high accelerating voltage (1,000 kV) enables bulk samples a few micrometers in thickness to be studied which minimizes surface effects^19^. The observations are then compared with MgH_2_ decomposition in thin-samples (tens of nm) with length scale analogous to nano-powders.

Figure 1 shows a TEM image and electron diffraction patterns, observed at an acceleration voltage of 300 kV, from a MgH_2_ particle produced by hydriding a Mg-Ni-based H-storage alloy. They show the presence of MgH_2_ and also Mg phase as hexagonal shaped Mg grains of around 30 to 60 nm size within the MgH_2_ matrix. From the low indexed electron diffraction patterns from MgH_2_ [101]* and Mg [001]* taken from the same selected area diffraction method, diffraction spots of (12-1) from MgH_2_ and (−210) from Mg overlap. That means the crystallographic orientation relationships (ORs) between MgH_2_ and Mg are MgH_2_ [101]*//Mg [001]* (beam direction) and MgH_2_ (12-1)//Mg (−210) (plane) as well as MgH_2_ (101)//Mg (002) since MgH_2_ [101]* and Mg [001]* are perpendicular to MgH_2_ (101) and Mg (002). The differences of lattice spacings between MgH_2_ (12-1) and Mg(−210) as well as between MgH_2_ (101) and Mg (002) are both about 4%. This crystallographic orientation would represent a low strain energy situation facilitating the hydriding phase transformation from Mg to MgH_2_. Also Figure 1 shows the hexagonal facets of the Mg grains have (100) and (1–10) habit planes within MgH_2_. Schober et.al.^21^ reported the relationships as MgH_2_ [001]*//Mg [−1–10] * and MgH_2_ (200)//Mg (002), which has a 15.5% lattice mismatch. Paik et. al.^18^ measured MgH_2_ [001]*//Mg [−210]* and MgH_2_ (−110)//Mg (002), which has a 18.4% mismatch.

Note that the sample in Figure 1 was nominally fully-hydrided and, therefore, it is necessary to consider why a small volume fraction of nano-sized Mg grains remained in the MgH_2_ matrix. In the supporting data, Figure S4 shows scanning electron microscopy (SEM) images prepared from samples quenched at selected times (e.g. 5, 8 and 20 hours) during hydrogen absorption at 340°C and 1 MPa. A large number of small MgH_2_ nuclei formed around the Mg dendrite in the early stages of hydrogenation and grew into the Mg phase with time. The cracking of the growing MgH_2_ phase in Figure S4 (b) and (c) is associated with the release of strain energy caused by the large volume change of transformation (30.4% at 340°C^22^). Even after a prolonged period of hydrogenation (e.g. 20 hours in Figure S4 (c)), small islands of the Mg phase are still retained in the MgH_2_ matrix. This is likely due to the impingement of growing MgH_2_ effectively providing a barrier against further hydrogen diffusion because the coefficient of hydrogen diffusion in MgH_2_ is at least three orders of magnitude less than that in Mg^23^, and may also be due to strain energy retarding growth as MgH_2_-Mg interfaces grow into the last small Mg islands surrounded by MgH_2_ (e.g. Figure 1). Therefore, a key finding in this work is that some Mg phase is retained after a ‘full' hydrogenation cycle. Importantly, it is expected that some Mg will be retained also at the end of recharging of industrial H-storage systems based on MgH_2_ bulk powder. This has a significant effect on dehydriding mechanisms as shown in the next sections.

In-situ TEM observations were performed on a ~2 μm particle of bulk powder at an acceleration voltage of 1,000 kV with a heating holder and a high resolution video recorder. The temperature at the TEM sample grid over the time of observation is in the supporting data (Figure S1). The average heating rate is approximately 13°C/min from 28 to 455°C. Concerns that the insulation effects between the TEM sample grid and the specimen may render the measured sample temperature inaccurate can be alleviated by comparing the differential scanning calorimetry (DSC) and TEM data. A comparison of the in-situ hydrogen release observations in the Movie S1, shows the MgH_2_ to Mg phase transformation is completed at around 420°C and the DSC experiments in Figure S5 show that the hydrogen release peak temperature was 423°C at a similar heating rate of 15°C/min.

Figure 2(a)–(d) shows selected still frame TEM images taken at the temperatures of (a) 300°C, (b) 420°C, (c) 430°C and (d) 455°C (the observation video is available in Movie S1). Several bright grains were observed in the sample in the low temperature range up to 300°C, which correspond to Mg grains, similar to those shown in Figure 1. Those Mg grains subsequently grow and coalesce, with increasing temperature (Figure 2). There are several large Mg grains at 455°C, which are clearly shown in the low magnification image in Figure 2(e). These events in the bulk sample correspond well with the DSC shown in the Figure S5 and corresponding Synchrotron XRD data under air and 0.1 MPa conditions in Figure S3^12^. This in-situ TEM result is direct evidence that dehydriding of ~2 μm bulk MgH_2_ particles occurs by the growth of multiple pre-existing Mg grains within a MgH_2_ matrix.

A volume change (shrinkage) of around 30% occurs during hydrogen release associated with the phase transformation from MgH_2_ to Mg (30.4% at 340°C reported by Ono et al^22^), and from Figure 2 and Movie S1, we can see some slight shrinkage of the bulk MgH_2_ particle (around 2 micrometers diameter) and Mg grains in the bulk MgH_2_ particle with concave shape at 455°C where hydrogen release had stopped. However, there was an absence of any void formation within the bulk particle. It is likely the strain associated with volumetric contraction is accommodated by deformation (including contraction in the transverse direction). At the temperatures involved this deformation appears to be more favourable than the nucleation and growth of porosity, which requires substantial energy.

For comparison, in-situ TEM was performed at 200 kV TEM (Figure 3 and Movie S2). In this case, the thickness in the observation area is a few tens of nanometers as required for imaging at 200 kV and is at the edge of the sample. Figure 3 shows selected still frame TEM images from the in-situ video in Movie S2 taken at the temperatures of (a) 50°C, (b) 150°C, (c) 250°C, and (d) 380°C. Mg grains form at the thinnest edge of the sample, and progress toward the inside of the MgH_2_ sample at ~150°C. This result indicates the mechanism of hydrogen release in this thin section of the sample is quite different from the bulk sample results obtained with high-voltage TEM in Figure 2 and Movie S1. Also, the hydrogen release temperature is much lower than that obtained by high voltage TEM observations (Figure 2 and Movie S1). The influence of conventional 200 kV TEM on dehydriding mechanisms includes, (1) the thin (less than 100 nm) sample thickness allowing surface effects to have a disproportionate effect, and (2) the inelastic incident beam interaction with the sample atoms being stronger than at high voltage and, as a result, more pronounced ‘electronic excitation' occurs.

From the direct in-situ observations of hydrogen release behavior from the bulk (thick) sample presented in this work, the observations are schematically shown in Figure 4(a), which is closest to the multiple ‘nucleation and growth' model^3^, with the important difference that small (tens of nm) Mg grains were pre-existing in the sample (Figure 1) and, therefore, nucleation was not a pre-requisite for the transformation. In this case, the transformation is growth controlled, where hydrogen atoms diffuse from MgH_2_ with the driving force for grain boundary movement being derived from the free energy difference between atoms in adjacent grains. Also from Figure 2(e), several areas near the grain surface have remained as MgH_2_ and oxide, indicating that even at 455°C, some volume still contains hydrogen as the MgH_2_ phase. The existence of both Mg and MgH_2_ phases after both charging and discharging is likely to play an important role in hydriding/dehydriding kinetics of bulk powder. The effect of pre-existing Mg grains on the dehydrogenation of MgH_2_ was reported by Tanniru et. al.^24^, using scanning electron microscopy. They concluded that the nucleation barrier for hexagonal close-packed Mg plays an important role in establishing the hydrogen release temperature. When the magnesium powders are hydrogenated such that the surface is completely covered by the hydride phase, the desorption temperature is found to be high, owing to the energy required for the nucleation of the Mg on the surface. Antisari et. al.^25^ have confirmed the difficulty of Mg nucleation in MgH_2_, and the presence of some retained Mg phase is likely to accelerate dehydriding kinetics. If there were no pre-existing Mg grains, surface nucleation would be easier than nucleation within the volume due to the volume change. However, in the presence of pre-existing Mg grains within the volume, growth from within is more favourable than nucleation and growth from the surface.

The thin sample (tens of nm thick) studied by conventional TEM at 200 kV in Figure 3 and Figure 4(b) may reflect the behavior of fine nano-powders as well as election beam heating effect due to the inelastic incident beam interactions with the samples^19^. In this case, the formation of Mg at the sample edge followed by growth into the MgH_2_ is consistent with ‘shrinking core' models, where nucleation and growth of Mg phase happen from the edge/surface of the sample. Based on ‘shrinking core' models, Ouyang et. al.^26^ found the hydriding/dehydriding process in nano-grained (around 30 nm in diameter) of Mg is catalyzed by the combination of in situ formed extremely fine CeH_2_/CeH_2.73_ and Ni to Mg/MgH_2_. The experiments performed were not isothermal and did not enable a comparison of the kinetics of hydrogen transportation through the surrounding MgH_2_ phase between the thin and bulk samples. While the overall thickness of the thin samples will be the dominant geometric factor influencing the desorption rate the spatial distribution of any pre-existing Mg nuclei and in particular the distance of these nuclei from the free surface will play an important role in determining the desorption rate of the bulk samples.

From in-situ TEM imaging, we conclude that the hydrogen release mechanism from bulk (2 μm) MgH_2_ particles is based on the growth of multiple pre-existing Mg grains (crystallites within the MgH_2_ matrix) present due to the difficulty of fully transforming all Mg during a hydrogenation cycle whereas, in thin samples analogous to nano-powders, dehydriding occurs by a ‘shrinking core' mechanism.

## Methods

### MgH_2_ sample preparation

To prepare samples for hydrogen absorption they were machined to fine chips in air using a drill press. The unreacted chips were of the order of 0.1 mm thick and a few mm in length. Hydrogen sorption was conducted using an automated gravimetric testing apparatus PCTM-5000A (Technosystem Ltd., Japan)^27^ using laboratory grade high purity H_2_ gas (99.98% purity). The apparatus levitates approximately one gram of the sample material and directly records the weight change using a balance. The details of this machine are described elsewhere^6^,^27^. The samples were ‘hydrided', under conditions of 2 MPa, 350°C for 20 hours. During hydriding, MgH_2_ and Mg_2_NiH_4_ formed and significant cracking occurred, reducing the particle size to a few μm. All TEM results presented in the main paper are from powder particles containing MgH_2_ and no discernable Mg_2_NiH_4_.

Sample preparation for high voltage (1,000 kV) TEM involved selecting single particles of MgH_2_ from the reaction product and loading them on a temperature controlled TEM sample holder with a 3 mm diameter grid for high temperature use. Note that particles were examined without any further mechanical/chemical preparations which would be difficult using conventional TEM with 200 kV.

Conventional 200 kV TEM was also performed for comparison with the 1,000 kV experiments and to explore the influence of sample thickness and beam heating on dehydriding mechanisms. To produce thin samples for conventional TEM, MgH_2_ particles were selected from the hydride powder and fractured in an agate mortar producing fragments with edges a few tens of nanometers in thickness.

### TEM observations

For the detailed crystallography of Mg and MgH_2_ and their relative orientation relationships, a transmission electron microscopy (TEM), JEM-3200FSK (JEOL, Japan) at acceleration voltage of 300 kV with an Omega filter was used. In-situ TEM observations were performed using a JEM-1000 (JEOL, Japan) at an acceleration voltage of 1,000 kV with an EM-HSTH (JEOL, Japan) heating holder and high resolution video recorder.

For comparison with conventional in-situ TEM observations, we used a JEM-2100HCLM (JEOL, Japan) at an acceleration voltage of 200 kV with a Model 652 (GATAN, U.S.A.) double tilt heating holder.

## Author Contributions

K.N. contributed to the planning of all experiments, participating TEM and Synchrotron XRD experiments and writing the article. X.Q.T. contributed to the discussion and conducted DSC and SEM experiments and Synchrotron XRD data analysis. T.Y. contributed to the discussion and TEM experiments and analysis. E.T. contributed to the discussion and TEM experiments and analysis. S.D.M. contributed to the planning of all experiments, discussion, and writing the article. C.M.G. contributed to the interpretation, discussions, and writing the article. K.Y. contributed to the planning of all experiments, TEM data analysis and discussion. S.M. contributed to the planning of all experiments, TEM data analysis, discussion, and writing the article.

## Supplementary Material

Supplementary Information Movie S1

Supplementary Information Movie S2

Supplementary Information Supplementary Materials

## Figures and Tables

**Figure 1:**
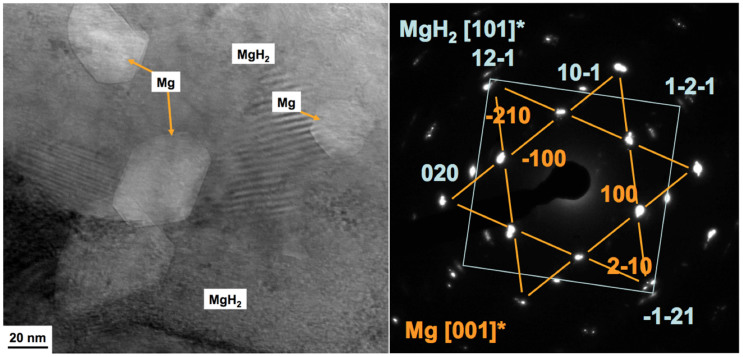
A TEM image and selected area electron diffraction patterns from Mg and MgH_2_ phases in a nominally fully hydrogenated sample.

**Figure 2:**
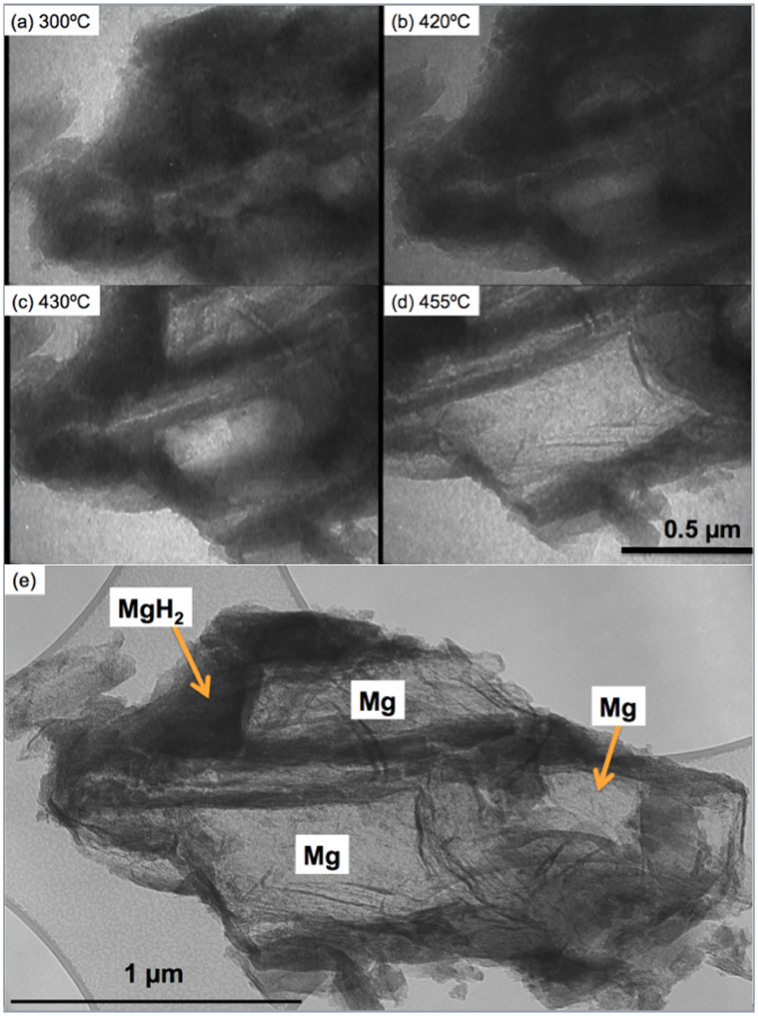
Selected still frame TEM images from in-situ video of high voltage (1,000 kV) TEM of a ~2 μm bulk MgH_2_ particle taken at (a) 300°C, (b) 420°C, (c) 430°C and (d) 455°C, and (e) a low magnification bright field image of the sample (a single bulk powder particle) at 455°C.

**Figure 3:**
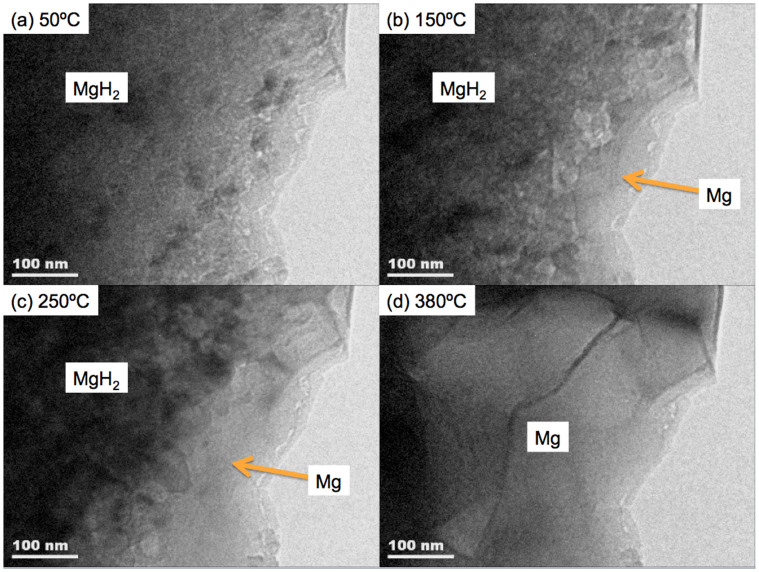
Selected still frame TEM images from in-situ video of conventional (200 kV) TEM through a thinned region (a few tens of nm) of a MgH_2_ particle taken at (a) 50°C, (b) 150°C, (c) 250°C, and (d) 380°C.

**Figure 4:**
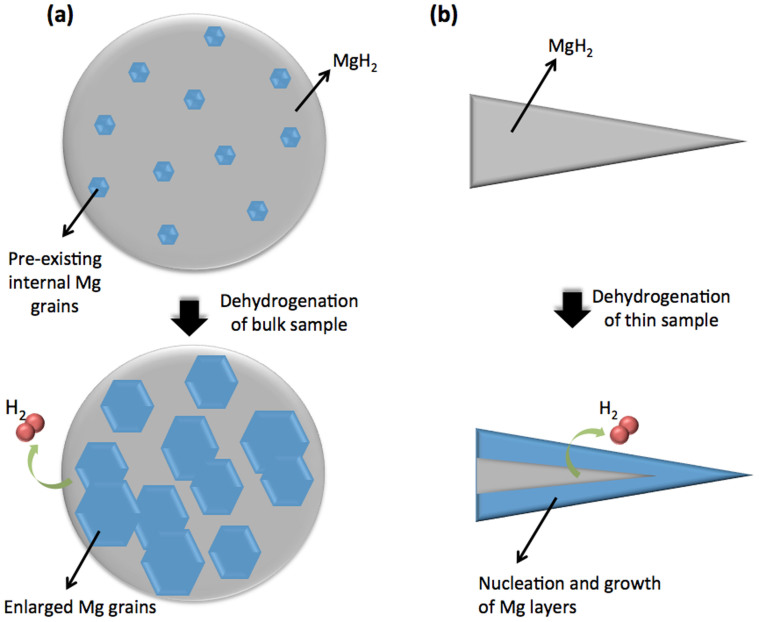
Schematic hydrogen release mechanisms from a MgH_2_ grain: (a) multiple ‘nucleation and growth' model for bulk MgH_2_ grains and (b) ‘shrinking core' model for thin MgH_2_ TEM samples.
