# Supplementary material for: Evidence of the hydrogen release mechanism in bulk MgH2
Source: Sci Rep. 2015 Feb 13;5:8450. doi: 10.1038/srep08450 (PMC4327414; doi:10.1038/srep08450)
Supplement: Supplementary Information — Supplementary Materials [file srep08450-s3.pdf]

Supplementary Materials

Evidence of the hydrogen release mechanism in bulk MgH_2

Kazuhiro Nogita[1, 2, *], Xuan Q. Tran[1], Tomokazu Yamamoto[2], Eishi Tanaka[2], Stuart D. McDonald[1], Christopher M. Gourlay[3], Kazuhiro Yasuda[2] and Syo Matsumura[2]

[1] Nihon Superior Centre for the Manufacture of Electronic Materials, School of Mechanical and Mining Engineering, The University of Queensland, Brisbane, QLD 4072, Australia
[2] Department of Applied Quantum Physics and Nuclear Engineering and The Ultramicroscopy Research Center, Kyushu University, Fukuoka, 819-0395, Japan
[3] Department of Materials, Imperial College, London. SW7 2AZ. UK

correspondence to: k.nogita@uq.edu.au

Contents

1. Mg alloy sample preparations and chemical compositions
2. TEM observations
3. Differential scanning calorimetry (DSC) of dehydriding reaction
4. In-situ synchrotron powder XRD of dehydriding reaction
5. Analysis of initial hydriding by SEM observations after interrupted hydriding
6. Analysis of the dehydriding transformation by combining DSC and XRD results

Supplementary References

List of Table, Figures and Movies

Table S1. Chemical composition of the sample measured by ICP-AES (wt%).

Fig. S1. Temperature profile during the in-situ observation by high voltage 1,000kV TEM.

Fig. S2. Temperature profile during the in-situ observation by conventional 200kV TEM.

Fig. S3. Development of phase fraction with temperature during desorption by heating under 0.1MPa air. [1] Measurements are based on Rietveld refinement of *in-situ* synchrotron XRD data. Note that the left hand y-axis spans 0-100 mol% and the right hand y-axis spans 0-10 mol%.

Fig. S4. SEM characterisation of partially hydrided samples after (a) 5 hours, (b) 8 hours and (c) 20 hours of hydrogenation.

Fig. S5. Typical DSC result of hydrogen desorption during heating at 15°C/min.

Movie S1. In-situ TEM observations by JEM-1000 (acceleration voltage is 1,000 kV) with an EM-HSTH heating holder and high resolution video recorder (8x speed).

Movie S2. In-situ TEM observations by JEM-2100HCLM (acceleration voltage is 200 kV) with GATAN Model 652 double tilt heating holder (8x speed).

1. Mg alloy sample preparations and chemical compositions

Detailed chemical compositions of the sample measured by Inductively Coupled Plasma - Atomic Emission Spectrometry (ICP-AES) is in Table S1. A processing route described previously [1,2] was used to generate a non-pyrophoric material that may be produced in large quantities at comparatively low cost and can be handled in air. The alloys investigated were based on the hypoeutectic Mg - 14 wt%Ni composition. Alloys were produced by first melting industrial purity magnesium under an SF_6 atmosphere in an electric resistance furnace. Industrial grade nickel powder was then added and stirred into the melt, and the melt was held for 30 minutes at 750°C to ensure full dissolution of the addition. Following this, 1,000 ppm of elemental Na was added to the melt. After homogenisation, the liquid was cast into cylindrical steel moulds preheated to 250°C with cavity dimensions of diameter 20 mm and height 200 mm. The resulting casting contained (Mg) dendrites and (Mg)-Mg_2Ni eutectic.

2. TEM observations

Figure S1 shows the temperature profile for the in-situ observation of the bulk sample. In-situ video (8x speed) can be found in Movie S1. A sample (MgH_2) particle was measured at room temperature, then heated from 25 to 460ºC maintaining each temperature to find the hydrogen release behaviour by analysing the phase changes. The beam-induced heating of hydride and dehydrided phases remains small at the acceleration voltage of 1,000kV, allowing for proper observations of the hydride [3].

Figure S2 shows the temperature profile for the in-situ observation of the thin sample. In-situ video (8x speed) can be found in Movie S2. It is noted that the thickness of the observation area is a few tens of nanomenters at the edge of the samples.

3. Differential scanning calorimetry (DSC) of dehydriding reaction

The characteristic temperatures of the hydrogen desorption reaction were examined by differential scanning calorimetry (DSC) (Mettler Toledo DSC 1, Switzerland). Samples were less than 10 mg and compacted into an aluminium pan and experiments were performed at a heating rate of 15°C/min up to a maximum temperature of about 450°C. The experiments were carried out under atmospheric pressure with an inert Nitrogen gas flow at a rate of 50 ml/min.

4. In-situ synchrotron powder XRD of dehydriding reaction

For *in-situ* X-ray Powder Diffraction (XRD) experiments, the powdered sample was loaded into a quartz capillary of 700 µm diameter within a gas flow cell and measured at the Powder Diffraction beamline at the Australian Synchrotron facility. The flow cell was connected to a nitrogen gas flow line with temperature controlled via a hot air blower. *In-situ* XRD experiments were then conducted while the sample was simultaneously

pressurized under ambient air and heated at various pre-set temperatures ranging from 220°C to 500°C. The ramp rate of temperature was 100°C/min. Each data point was taken based on an average data recording over 10 minute periods. The Rietveld refinement method was used for data analysis with a summary of the result presented in Figure S3. More detailed descriptions of the experimental procedure and data analysis can be found elsewhere [1].

5. Analysis of initial hydriding by SEM observations after interrupted hydriding

In order to understand microstructure development during hydriding, the hydriding process was interrupted and the resulting microstructures were characterized by Scanning Electron Microscopy (SEM). The samples were prepared by quenching at selected timeframes (e.g. 5, 8 and 20 hours) during hydrogen absorption at 340°C and 1 MPa in PCTM-5000A. When quenching, the pressure was still maintained while the material was being cooled down to room temperature to prevent undesired desorption of the samples.

Microstructural characterisation was carried out using a SEM JEM6610 (JEOL, Japan) in backscatter electron mode. It is clearly evidenced from Figure S4 that the mechanism of nucleation and growth of the hydride phase has taken place where a large number of small MgH_2 nuclei formed around the Mg dendrite in the early stages of hydrogenation and grew into larger sizes. It is observed that even after a prolonged period of hydrogenation (e.g. after 20 hours), small islands of Mg phase still remain. The reasons for this are discussed in the main paper.

6. Analysis of the dehydriding transformation by combining DSC and XRD results

Figure S5 shows a typical DSC response at 15°C/min. The first endothermic peak that occurs at 240-253°C is associated with the well-known allotropic phase transition of Mg_2NiH_4 from its low temperature (LT) tetragonal form to the high temperature (HT) cubic polymorph as previously reported by other authors: 242°C according to [4], 250°C according to [5] or in the range of 220-245°C as reported by [6]). This is also in a good agreement with our XRD result under air, which shows T_{Tr} = 248˚C (Figure S3). The DSC response then shows a hydrogen release temperature at around 423°C. This temperature corresponds well to the temperature obtained by in-situ high voltage TEM observations shown in Figure 3 and Movie S1 at the heating rate of 13°C/min.

The XRD result in Figure S3 shows that the decomposition reactions for MgH_2 and (HT) Mg_2NiH_4 are overlapping and occur in the range 320-412°C. Based on this, it is likely that the second endothermic peak in the DSC curves (Figure S5) at about 395-434°C also contains these two reactions overlapping. Since the final reaction product contains ~94% Mg and ~6% Mg_2Ni (Figure S3), the majority of the peak is associated with Mg formation. For comparison, literature reported results for hydrogen desorption of MgH_2 are in the range of 320-450°C dependent on the presence of various catalysts (e.g. $NaNH_2$ [7], Fe [8], $FeCl_3$ [9] or graphite [10], respectively) and heating rate. These values are well above the equilibrium temperature of T_{eq}= 287°C calculated for MgH_2 dehydriding in the presence of Mg_2Ni under 0.1MPa of hydrogen according to Reilly et al [11]. Similarly, the

desorption temperature for Mg_2NiH_4 was reported as 320°C [5], or 327°C [4] under 0.1MPa of hydrogen, well above the equilibrium temperature for Mg_2NiH_4 desorption at 253°C as calculated from the Van't Hoff's equation [11].

Note that the in-situ TEM results in the main paper are from individual powder particles of MgH_2 that did not contain any discernable Mg_2NiH_4.

Supplementary References

1 Nogita, K., McDonald, S. D., Duguid, A., Tsubota, M. & Gu, Q. F. Hydrogen desorption of Mg-Mg2Ni hypo-eutectic alloys in Air, Ar, CO2, N2 and H2. *J. Alloys Compd.* **580** S140-S143 (2013).

2 Nogita, K. *et al.* Engineering the Mg-Mg_2Ni eutectic transformation to produce improved hydrogen storage alloys. *Int. J. Hydrogen Energy* **34**, 7686-7691 (2009).

3 Danaie, M. & Mitlin, D. TEM analysis and sorption properties of high-energy milled MgH2 powders. *J. Alloys Compd.* **476**, 590–598 (2009).

4 J. Čermák, L. K., B. David. Hydrogen diffusion in Mg2NiH4 intermetallic compound. *Intermetallics* **16**, 508-517 (2008).

5 Polanski, M. *et al.* Mg2NiH4 synthesis and decomposition reactions. *International Journal of Hydrogen Energy,* **38**, 4003-4010 (2013).

6 Gavra, Z., Mintz, M. H., Kimmel, G. & Hadari, Z. Allotropic transitions of magnesium nickel hydride (Mg2NiH4). *Inorg. Chem.* **18**, 3595 (1979).

7 Milošević, S. *et al.* Hydrogen desorption properties of MgH2 catalysed with NaNH2. *Int. J. Hydrogen Energy* **38**, 12223-12229 (2013).

8 Antisari, M. V. *et al.* Scanning electron microscopy of partially de-hydrogenated MgH2 powders. *Intermetallics* **17**, 596–602 (2009).

9 Ismail, M. Influence of different amounts of FeCl3 on decomposition and hydrogen sorption kinetics of MgH2. *Int. J. Hydrogen Energy* **39**, 2567-2574 (2014).

10 Montone, A. *et al.* Microstructure, surface properties and hydrating behaviour of Mg-C composites prepared by ball milling with benzene. *Int. J. Hydrogen Energy* **31**, 2088-2096 (2006).

11 Reilly, J. J. & R. H. Wiswall, J. The Reaction of hydrogen with alloys of magnesium and nickel and the formation of Mg2NiH4. *Inorg. Chem.* **7**, 2254-2256 (1968).

Table S1
Chemical composition of the sample measured by ICP-AES (wt%).

Sample	Mg	Ni	Na	Ca	Eu	Fe	Cu	Zr	Al
Mg-14wt%Ni	Bal.	14.3	0.09	< 0.005	< 0.005	< 0.01	< 0.005	< 0.005	< 0.01

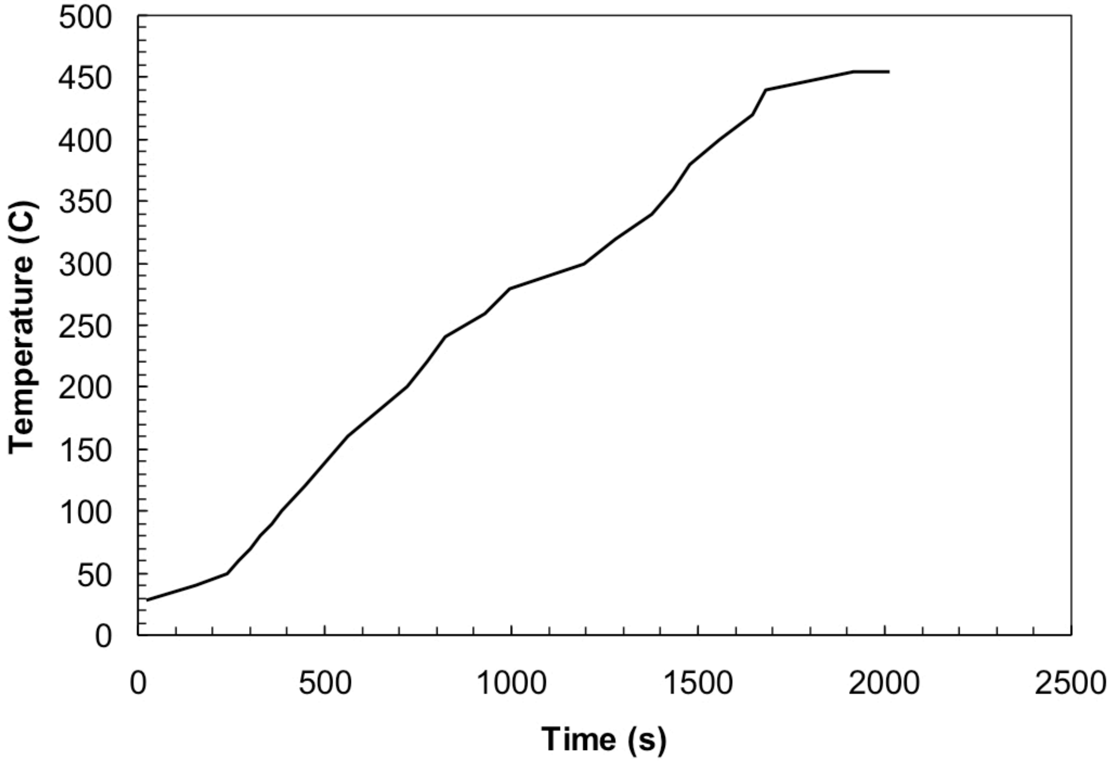

Fig. S1

Temperature profile during the in-situ observation by high voltage 1,000kV TEM.

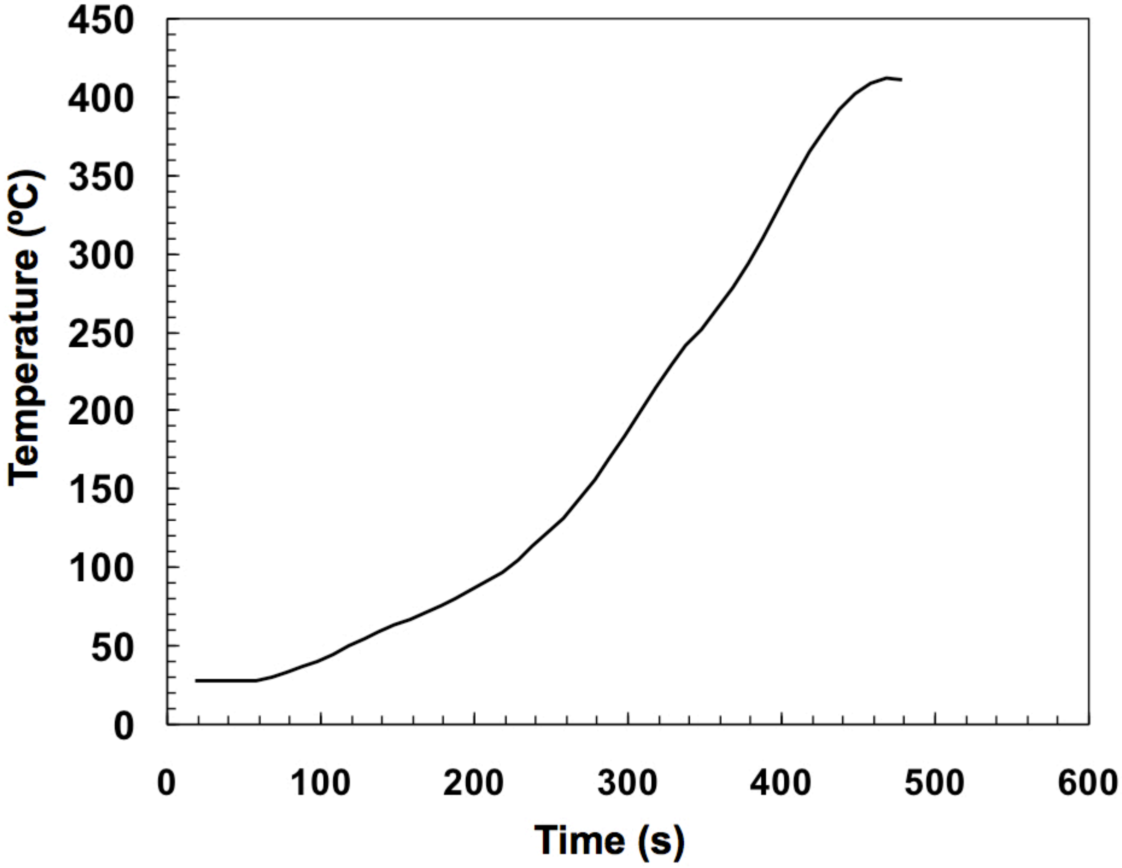

Fig. S2
Temperature profile during the in-situ observation by conventional 200kV TEM.

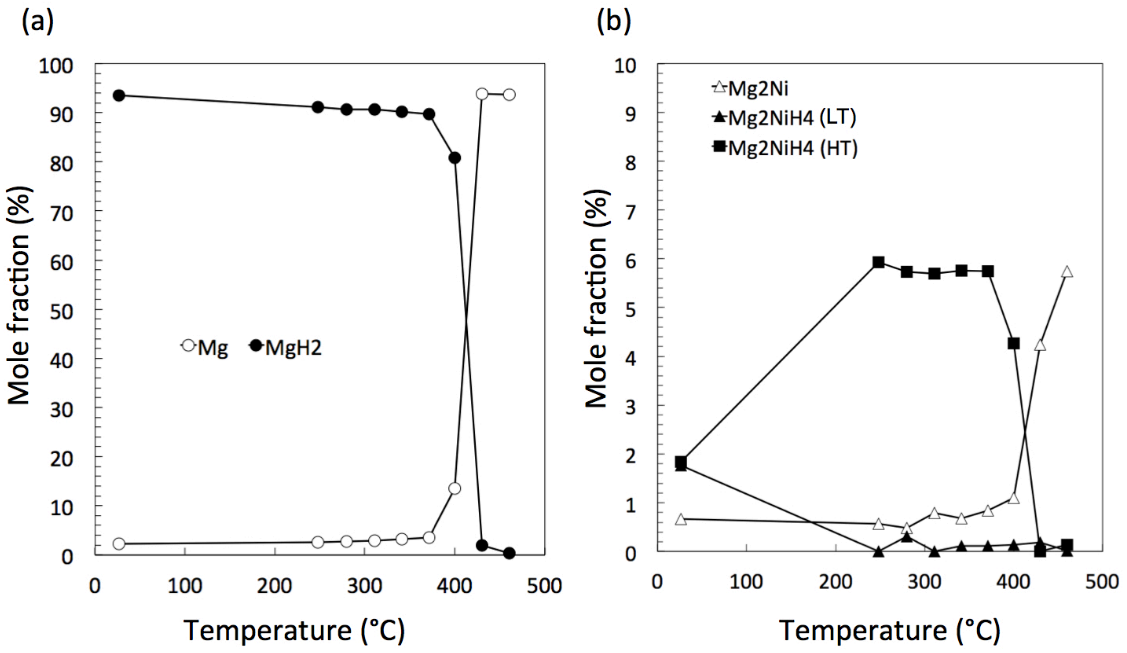

Fig. S3

Development of phase fraction with temperature during desorption by heating under 0.1MPa air. [1] Measurements are based on Rietveld refinement of *in-situ* synchrotron XRD data. Note that the left hand y-axis spans 0-100 mol% and the right hand y-axis spans 0-10 mol%.

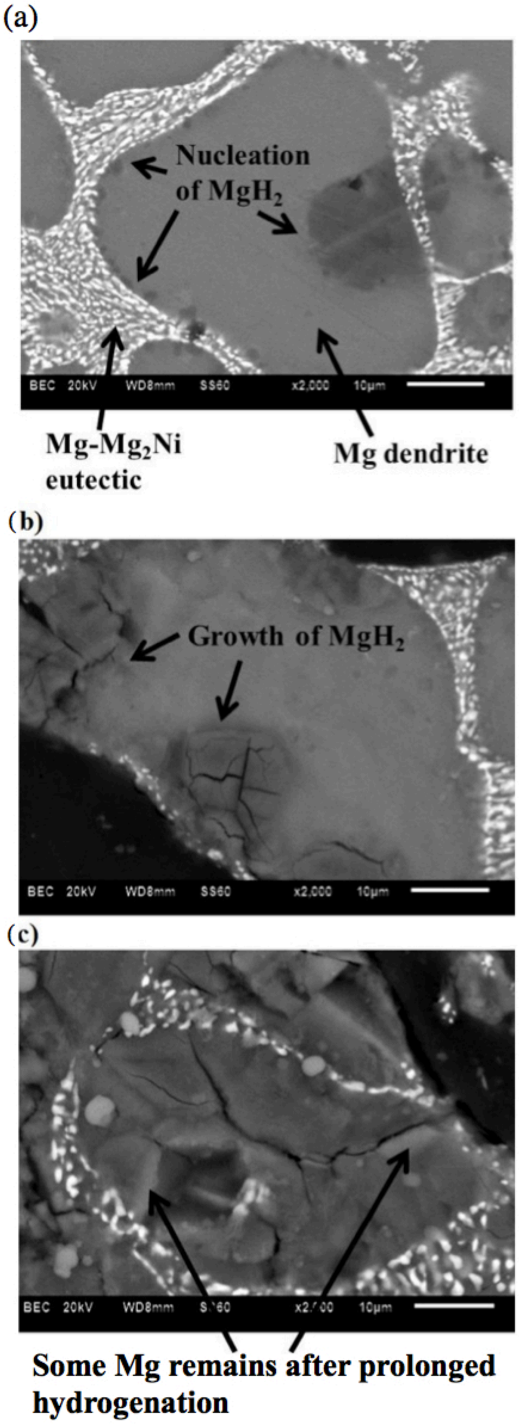

Fig. S4

SEM characterisation of partially hydrided samples after (a) 5 hours, (b) 8 hours and (c) 20 hours of hydrogenation.

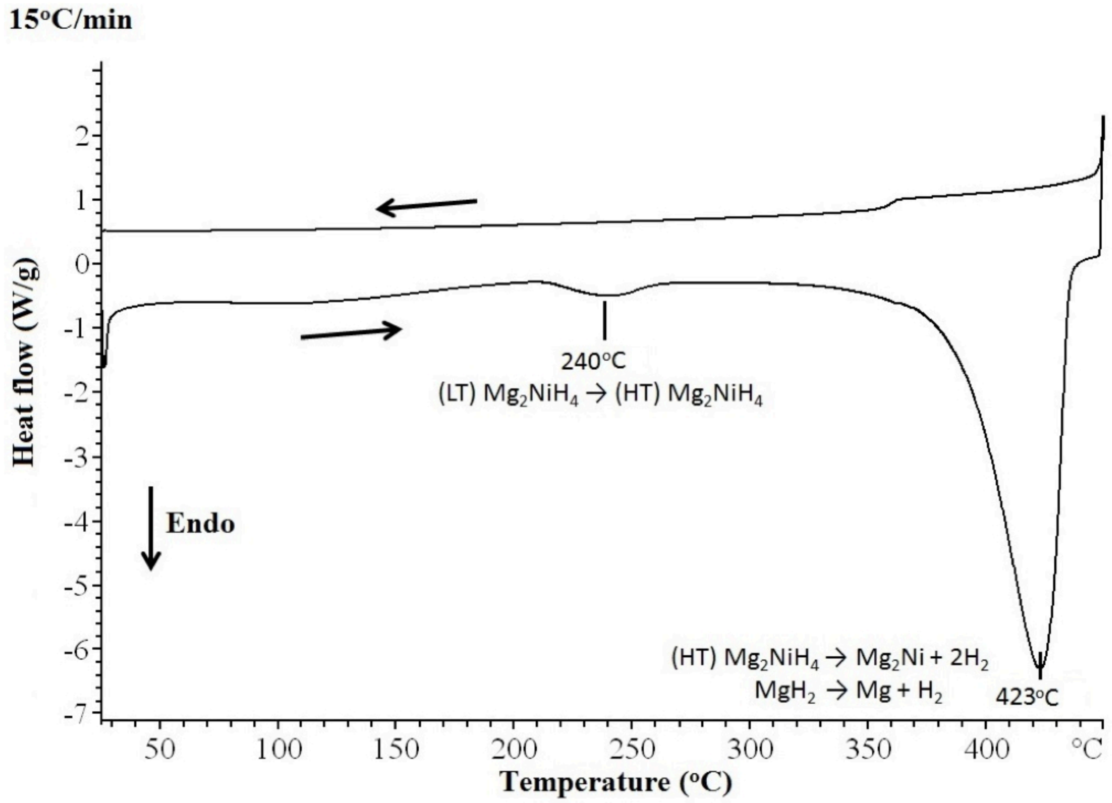

Fig. S5

Typical DSC result of hydrogen desorption during heating at 15°C/min.
